# COVID-19 and Its Psychological Effects on the Elderly Population

**DOI:** 10.1017/dmp.2020.245

**Published:** 2020-07-15

**Affiliations:** Department of Social Work, University of Social Welfare and Rehabilitation Sciences, Tehran, Iran; Department of Counseling, University of Social Welfare and Rehabilitation Sciences, Tehran, Iran

**Keywords:** coronavirus, elderly population, psychological effects

In late December 2019, a cluster of patients was admitted to hospitals with an initial diagnosis of pneumonia of an unknown etiology. These patients were epidemiologically linked to a seafood and wet animal wholesale market in Wuhan, Hubei Province, China.^1^ The virus was identified as a coronavirus and officially named *severe acute respiratory syndrome coronavirus 2 (SARS-CoV-2)*.

Coronaviruses (CoV) are a large family of viruses that cause illness ranging from the common cold to more severe diseases. The novel coronavirus (nCoV) is a new strain that has not been previously identified in humans.^2^

The symptoms of *coronavirus disease* (COVID-19), the disease caused by infection with SARS-CoV-2, appear after an incubation period of approximately 5.2 days. The period from the onset of COVID-19 symptoms to death ranged from 6 to 41 days with a median of 14 days. This period is dependent on the age of the patient and status of the patient’s immune system. It was shorter among patients > 70 years old compared with those under the age of 70.^3^

As the COVID-19 pandemic rapidly sweeps across the world, it is inducing a considerable degree of fear, worry, and concern in the population at large and among certain groups in particular, such as older adults, care providers, and people with underlying health conditions.^4^

In public mental health terms, the main psychological impact to date is elevated rates of stress or anxiety. But as new measures and impacts are introduced – especially quarantine and its effects on many people’s usual activities, routines, or livelihoods – levels of loneliness, depression, harmful alcohol and drug use, and self-harm or suicidal behavior are also expected to rise.^4^

As countries are affected by COVID-19, the elderly population will soon be told to self-isolate for “a very long time” all over the world, although it is well known that social isolation among older adults is a “serious public health concern” because of their heightened risk of cardiovascular, autoimmune, neurocognitive, and mental health problems. Santini and colleagues recently demonstrated that social disconnection puts older adults at greater risk of depression and anxiety.^5^

If health ministers instruct elderly people to remain home, have groceries and vital medications delivered, and avoid social contact with family and friends, urgent action is needed to mitigate the mental and physical health consequences.^6^

Self-isolation will disproportionately affect elderly individuals whose only social contact is out of the home, such as at daycare venues, community centers, and places of worship. Those who do not have close family or friends, and rely on the support of voluntary services or social care, could be placed at additional risk, along with those who are already lonely, isolated, or secluded.^7,8^

Regarding older people and also those with underlying health conditions, having been identified as more vulnerable to COVID-19, and to be told that they are very vulnerable, can be extremely frightening and very fear-inducing.^4^ The psychological impacts for these populations can include anxiety and feeling stressed or angry.^9^ Its impacts can be particularly difficult for older people who may be experiencing cognitive impairment or dementia. Some older people may already be socially isolated and experiencing loneliness, which can worsen mental health.^10^

On a positive note, there are many things that older people can initiate themselves or with the support of a caregiver, if needed, to protect their mental health at this time. These include many of the strategies that we are advocating across the entire population, such as undertaking physical activity, keeping to routines or creating new ones, and engaging in activities that give a sense of achievement.^11^ Maintaining social connections is also important. Some older people may be familiar with digital methods and others may need guidance in how to use them.^12^

Online technologies could be harnessed to provide social support networks and a sense of belonging, although there might be disparities in access to or literacy in digital resources.^12,13^ Interventions could simply involve more frequent telephone contact with significant others, close family and friends, voluntary organizations, or health care professionals or community outreach projects providing peer support throughout the enforced isolation. Beyond these, cognitive behavioral therapies could be delivered online to decrease loneliness and improve mental well-being.^14^

Any psychiatric/psychological intervention may be applied as in-patient/in-person or out-patient/out-person or teletherapy manner. Integrated psychiatrists, psychologists, general practitioners, crisis intervention specialists and social workers into teletherapy of patients, their caregivers/families, and medical staff have been recommended.^15^

This letter suggests the following recommendations for future interventions: (1) More attention needs to be paid to vulnerable groups, especially the elderly population; (2) accessibility to medical resources and the public health service system should be further strengthened and improved, particularly after reviewing the initial coping and management of the COVID-19 epidemic; (3) nationwide strategic planning and coordination for psychological first aid during major disasters, potentially delivered through telemedicine, should be established; and (4) a comprehensive crisis prevention and intervention system, including epidemiological monitoring, screening, referral and targeted intervention, should be built to reduce psychological distress and prevent further mental health problems among this population.^7^
